# Quality of Life in Newly Diagnosed Patients With *Parkin*-Related Parkinson's Disease

**DOI:** 10.3389/fneur.2020.580910

**Published:** 2020-12-18

**Authors:** Xin-Yue Zhou, Feng-Tao Liu, Chen Chen, Su-Shan Luo, Jue Zhao, Yi-Lin Tang, Bo Shen, Wen-Bo Yu, Chuan-Tao Zuo, Jian-Jun Wu, Zheng-Tong Ding, Jian Wang, Yi-Min Sun

**Affiliations:** ^1^Department of Neurology, National Clinical Research Center for Aging and Medicine, Huashan Hospital, Fudan University, Shanghai, China; ^2^Positron Emission Tomography (PET) Center, Huashan Hospital, Fudan University, Shanghai, China

**Keywords:** Parkinson's diasese, quality of life, *Parkin* (PARK2) gene mutation, newly diagnosed, early-onset, genetics

## Abstract

**Introduction:** Mutations in the *Parkin* gene are the most common cause of autosomal recessive early-onset Parkinson's disease (PD). However, little is known about the quality of life (QoL) in *Parkin*-related PD. Here, we investigated the patterns of QoL in newly diagnosed *Parkin*-related PD patients.

**Methods:** Newly diagnosed PD patients (diagnosis made within 12 months) who had an age of onset (AOO) below 40 and underwent a PD-related genetic testing, were recruited (*n* = 148). Among them, 24 patients carried bi-allelic variants in *Parkin* (PD-*Parkin*) and 24 patients did not have any known causative PD mutations, or risk variants (GU-EOPD). The clinical materials, relevant factors and determinants of QoL were analyzed.

**Results:** PD-*Parkin* patients had a younger AOO (*p* = 0.003) and longer disease duration (*p* = 0.005). After adjustment for AOO and disease duration, more dystonia (*p* = 0.034), and worse scores of non-motor symptoms including Beck depression inventory (BDI, *p* = 0.035), Epworth sleepiness scale (ESS, *p* = 0.044), and subdomains of depression/anxiety (*p* = 0.015) and sleep disorders (*p* = 0.005) in Non-motor symptoms questionnaire, were found in PD-*Parkin* comparing with GU-EOPD. PD-*Parkin* patients had poorer QoL (adjusted *p* = 0.045), especially in the mobility (adjusted *p* = 0.025), emotional well-being (adjusted *p* = 0.015) and bodily discomfort dimensions (adjusted *p* = 0.016). BDI scores (*p* = 0.005) and ESS scores (*p* = 0.047) were significant determinants of QoL in *PD-Parkin*.

**Conclusion:** Newly diagnosed PD-*Parkin* patients showed worse QoL. More depression and excessive daytime sleepiness predicted worse QoL. For clinicians, management of depression and excessive daytime sleepiness is suggested to better improve QoL in patients with *Parkin* mutations.

## Introduction

Parkinson's disease (PD), one of the most prevalent progressive neurodegenerative disorders, is characterized by motor dysfunctions such as bradykinesia, tremor and rigidity, as well as varieties of non-motor symptoms (NMSs) (1). Bi-allelic mutations in the *Parkin* gene are the most common cause of autosomal recessive early-onset PD (EOPD), accounting for 10.1% of patients with disease onset before the years of 40 in the Asian population (2). So far, genotypic and phenotypic characteristics of *Parkin*-related PD have been well-demonstrated in the literature. *Parkin*-related PD patients tend to have lower limb dystonia at onset, sleep benefits, marked response to levodopa, as well as mild progression (3–6).

Reduced quality of life (QoL) as an important aspect of PD is getting more and more attention, and the improvement in QoL has been increasingly acknowledged as a vital objective in the management of PD. A variety of studies have put their focus on the QoL of PD patients (7, 8), with some assessed the correlation between young age of onset (AOO) and poor quality of life (9, 10). However, scanty attention has been paid to QoL in monogenic forms of PD, including PD caused by *Parkin* mutations. Studies before have investigated determinants of quality of life in PD patients and found non-motor symptoms were the strongest predictor exhibiting negative impact on QoL (11). Depression and sleep disorders, in particular, excessive daytime sleepiness, were frequent non-motor symptoms and had a significant impact on QoL (12, 13). Nevertheless, the determinants of QoL in *Parkin*-related PD, and whether the non-motor symptoms also impaired the QoL significantly in these patients remain unknown.

Herein, we aimed to investigate the patterns of QoL in newly diagnosed PD patients with *Parkin* mutations comparing with the genetically undefined EOPD (GU-EOPD) patients. We chose the newly diagnosed PD patients to reduce the interference of PD medications and levodopa related motor complications on the QoL. Moreover, we analyzed the clinical data of PD-*Parkin* patients and further assessed the factors affecting the QoL in patients carrying bi-allelic *Parkin* mutations. Our study will offer clinicians further evidence for carrying out symptomatic management in *Parkin*-related PD for the best improvement of QoL.

## Materials and Methods

### Participants

A series of PD patients recruiting consecutively between June 2014 and December 2019 in our movement disorder clinic who met all the following criteria were investigated: (1) a PD diagnosis made within 12 months; (2) AOO <40 years; (3) PD genetic testing was received. The PD diagnosis was made based on the United Kingdom Parkinson's Disease Society Brain Bank criteria (14) or Movement Disorder Society Clinical Diagnostic Criteria for PD (15).

A total of 148 patients met the criteria. Participants with any pathogenic, likely pathogenic variants in PD causative genes except *Parkin*, heterozygous *GBA* variants*, LRRK2* risk variants (p.Gly2385Arg or p.Arg1628Pro), or heterozygous *Parkin* variants were then excluded. We also didn't include patients who harbored at least 2 heterozygous variants in *Parkin*, but without further testing in family members to determine the cis/trans phase of variants. Participants with insufficient clinical information were also ruled out. Forty-eight patients were included in the final analysis. Among them, there were 24 patients with compound heterozygous or homozygous variants in *Parkin* (PD-*Parkin* group) (Supplementary Table 2). The other 24 patients without any known causative PD mutations, or risk variants, were defined as the control group (GU-EOPD group) (Supplementary Figure 1).

All the participants in our study were from the Chinese Han population. They all signed informed consent in accordance with the Declaration of Helsinki before participation. The study was approved by the Human Studies Institutional Review Board, Huashan Hospital, Fudan University.

### Genetic Analysis

Genomic DNA samples were extracted from peripheral blood leucocytes of the participants (Qiagen, Germany). The genetic sequencing and analysis were carried out as previously reported (16). Briefly, a panel containing 49 PD associated genes was applied (Supplementary Table 1). The exons and flanking areas of the genes in the panel were selected as target regions. Samples were prepared as an Illumina sequencing library and were sequenced on the HiSeq2000 platform (Illumina, San Diego, CA). The variant screening protocol was as previously reported (16).

Multiplex ligation-dependent probe amplification (MLPA) was used to detect large deletions or duplications of exons or whole gene of common PD-causative genes including *SNCA, LRRK2, Parkin, PINK1, DJ1, PLA2G6, ATP13A2, UCHL1*, and *GCH1* by the kit of SALSA MLPA probemix P051-D1/P052-D1 Parkinson (MRC Holland).

The selected variants and detected large deletions or duplications were further tested in the family members of patients by Sanger sequencing or MLPA respectively if necessary and possible. The novel variants found in the patients were rated based on the guideline recommended by the American College of Medical Genetics (ACMG) (17).

The sequencing depth of target regions was 181.3x in average and the mean proportion of the target regions with at least 20x coverage was 96.91%. In total, 643 regions were sequenced. Among them, 13 (2.0%) regions had the depth of sequencing below 20x, mainly in GC-rich areas as described in our previous work (16). The coverage of most regions was 100%, and those with coverage under 100% were in the GC-rich regions (16).

### Clinical Assessments

Patients' demographic and clinical data were collected including sex, age, education year, AOO, disease duration, levodopa daily dosage (LEDD), usage of dopaminergic agonist, motor symptoms, motor complications, and non-motor symptoms as previously reported (16). Among these, the motor symptoms were assessed using the motor part of Unified Parkinson's Disease Rating Scale (UPDRS-III) and the Hoehn and Yahr scale (H&Y, range 0–5). The general condition of non-motor symptoms was determined using the Non-Motor Symptoms Questionnaire (NMSQ). The depression assessment was performed using the Beck Depression Inventory-2 (BDI-2). Excessive daytime sleepiness (EDS) was evaluated by the Epworth sleepiness scale (ESS), and rapid eye movement behavior disorders (RBD) were evaluated by the RBD screening questionnaire (RBDSQ); The global cognitive function was evaluated using the Mini-Mental State Exam (MMSE). Neuropsychological evaluations covering five domains of cognition were applied: Stroop Color-Word Test and Trail Making Test for executive function; Symbol Digital Modalities Test (SDMT) for attention; Rey-Osterrieth Complex Figure Test (CFT) and Clock Drawing task (CDT) for visuospatial function; CFT and Auditory Verbal Learning Test, AVLT) for memory; Verbal Fluency Test (VFT) and Boston Naming Test (BNT) for language. The quality of life was determined using the Parkinson's Disease Questionnaire-39 (PDQ-39), with higher scores presenting poorer life quality. It's worth pointing out that all the raters were blind to the genetic information of the participants, and whether the participant was aware of the genetic status was at random, as the clinical assessment was carried out before or after genetic testing randomly.

### Statistical Analysis

Descriptive data were expressed as mean ± SD. Significance was set at *p* < 0.05. The Student's *t*-test was applied in the analysis of continuous variables, while the difference in gender, motor subtype and dopaminergic agonist usage was compared by the Chi-squared-test. Analysis of covariance (ANCOVA) or logistic regression was applied to adjust for age of onset and disease duration depending on the types of variables. Furthermore, Pearson's correlation was applied to assess the correlation between QoL and clinical features, motor symptoms and non-motor symptoms. Clinical parameters with significant correlations with QoL were then included in the multiple linear regression model to investigate the main determinants of QoL in PD-*Parkin*. All analysis was performed using SPSS 22.0 (SPSS Inc., Chicago, IL).

## Results

### Demographic and Clinical Characteristics

Demographic and clinical characteristics of the two groups were compared in Table 1. The PD-*Parkin* group and GU-EOPD group did not differ regarding sex, age, and education years. A significantly younger AOO (*p* = 0.003) and longer disease duration (*p* = 0.005) were found in PD-*Parkin* group. Compared with the GU-EOPD group, the PD-*Parkin* group showed a higher prevalence of dystonia (*p* = 0.007), and the difference remained significant after adjustment for AOO and disease duration (*p* = 0.034). There was a trend that motor fluctuations were more common in patients with *Parkin* mutations, though not reaching the statistically significant level. As for PD medication, levodopa was the first choice in about half the patients, and other choices included dopaminergic agonist, benzhexol, and MAO-B (monoamine oxidase-B) inhibitors. The two groups showed no difference in LEDD, levodopa dosage, and dopaminergic agonist usage. No difference was observed in motor subtype, H&Y stage, and UPDRS III scores.

**Table 1:** Demographic and clinical characteristics of overall study population.

	**PD-*Parkin n* = 24**	**GU-EOPD *n* = 24**	**P_**1**_**	**P_**2**_**
Male%, *n*	50%, 12	66.67%, 16	0.242	
Age (year)	33.17 ± 6.74	35.25 ± 6.30	0.228	
Education (year)	11.52 ± 3.91	13.38 ± 3.74	0.104	
AOO (year)	27.08 ± 7.11	32.71 ± 4.89	0.003^**^	
Disease duration (month)	81.46 ± 77.47	31.25 ± 19.19	0.005^**^	
LEDD (mg/d)	287.54 ± 195.94	269.82 ± 261.10	0.791	
Levodopa dosage	137.50 ± 121.63	131.25 ± 194.52	0.890	
**Motor subtype**				
TD	1	4	0.156	
Indeterminate	6	3	0.267	
PIGD	17	17	1.000	
Dystonia (cases, %)	13, 54.17%	4, 16.67%	0.007^*^	0.034^*^
Motor fluctuations (cases, %)	6, 25.00%	1, 4.17%	0.188	0.051
Dyskinesias (cases, %)	2, 8.33%	0, 0%	0.245	NA
UPDRS III	26.83 ± 10.15	24.38 ± 12.82	0.465	0.697
H&Y	2.13 ± 0.54	1.88 ± 0.54	0.113	0.352
NMSQ	10.50 ± 6.50	6.70 ± 3.90	0.019^*^	0.088
**Domains**				
Digestive	2.94 ± 2.31	2.00 ± 1.53	0.332	0.557
Urinary	0.94 ± 0.80	0.94 ± 0.80	0.731	0.790
Sexual	0.61 ± 0.78	0.17 ± 0.38	0.027^*^	0.113
Cardiovascular	0.44 ± 0.62	0.22 ± 0.43	0.096	0.146
Memory	1.17 ± 0.99	0.56 ± 0.51	0.066	0.428
Hallucinations/delusions	0.61 ± 0.61	0.28 ± 0.46	0.168	0.608
Depression/anxiety	1.33 ± 0.69	0.44 ± 0.62	0.001^**^	0.015^*^
Sleep disorder	1.61 ± 1.3	0.72 ± 1.02	0.011^*^	0.005^**^
Miscellany	1.83 ± 1.20	1.33 ± 0.97	0.108	0.254
BDI	16.25 ± 9.59	8.52 ± 7.30	0.003^**^	0.035^*^
ESS	6.25 ± 3.50	3.26 ± 2.38	0.001^**^	0.044^*^
RBDSQ	3.79 ± 2.27	3.30 ± 2.40	0.478	0.939
MMSE	28.67 ± 1.34	28.30 ± 2.08	0.479	0.298
Rey-Osterrieth time	180.04 ± 106.58	182.43 ± 83.00	0.932	0.192
Rey-Osterrieth score	33.88 ± 1.92	33.30 ± 3.71	0.509	0.292
Rey-Osterrieth recall score	18.79 ± 6.80	18.74 ± 6.23	0.978	0.664
Stroop word color task (Part-C) time	69.92 ± 17.98	67.55 ± 24.47	0.708	0.661
Stroop word color task (Part-C)	47.21 ± 2.87	46.36 ± 7.66	0.617	0.702
Boston naming test	23.83 ± 4.14	24.64 ± 2.97	0.457	0.613
SDMT	47.58 ± 12.18	41.48 ± 12.03	0.091	0.162
AVLT-I	17.25 ± 4.49	17.48 ± 5.26	0.873	0.763
AVLT-T	29.63 ± 7.63	28.78 ± 9.25	0.735	0.949
Trail making task time	108.75 ± 46.72	118.70 ± 54.08	0.503	0.395
Verbal fluency test-1	18.25 ± 5.88	18.43 ± 5.33	0.911	0.927
Verbal fluency test-2	14.21 ± 6.59	17.65 ± 8.58	0.129	0.436
Verbal fluency test-3	16.38 ± 4.22	16.87 ± 4.51	0.699	0.808
Clock drawing task 30s	19.13 ± 5.60	20.52 ± 4.76	0.375	0.256
Dopaminergic agonist usage (cases, %)	2, 8.33%	7, 29.17%	0.064	

*P_1_, comparison between PD-Parkin and GU-EOPD. P_2_, comparison between PD-Parkin and GU-EOPD after adjustment for age of onset and disease duration*.

* p < 0.05;

** *p < 0.01. AOO, Age of onset; AVLT-I, Auditory verbal learning test short term memory; AVLT-T, Auditory verbal learning test total scores; BDI, Beck depression inventory; ESS, Epworth sleepiness scale; GU-EOPD, Genetically undefined early-onset Parkinson's disease; H&Y stage, Hoehn and Yahr stage; LEDD, Levodopa equivalent daily dosage; MMSE, Mini-mental state examination; NA, not applicable; NMSQ, Non-motor symptoms questionnaire; PIGD, postural instability/gait difficulty; PD, Parkinson's disease; RBDSQ, Rapid eye movement behavior disorders screening questionnaire; SDMT, Symbol digital modalities test; TD, tremor dominant; UPDRS III, Unified Parkinson's Disease Rating Scale part III*.

In terms of NMSs, though no statistical significance was found in NMSQ after adjustment, the subdomains of sleep disorder (*p* = 0.011; *p* = 0.005 after adjustment) and depression/anxiety (*p* < 0.001; *p* = 0.015 after adjustment) were more affected in the PD-*Parkin* patients comparing with the GU-EOPD patients. In addition, higher scores in ESS (*p* = 0.001, *p* = 0.044 after adjustment) and BDI scales (*p* = 0.003; *p* = 0.035 after adjustment) of PD-*Parkin* patients also gave the evidences that the PD-*Parkin* patients were more affected in sleep problems and depression. Since the usage of dopaminergic agonist and levodopa between the two groups were similar, the significant difference in ESS scores was less related to the medications. No difference was observed in MMSE and all the other neuropsychological evaluations.

### Quality of Life in PD Patients With *Parkin* Mutations and GU-EOPD

In the newly diagnosed PD patients, PD-*Parkin* group reported more reduced QoL compared with GU-EOPD group (*p* = 0.005; *p* = 0.045 after adjustment) (Table 2). PD-*Parkin* patients were more affected in subscales of mobility (*p* = 0.025), emotional well-being (*p* = 0.015), and bodily discomfort (*p* = 0.016) after adjustment for AOO and disease duration when comparing with GU-EOPD.

**Table 2:** Comparison of PDQ-39 between PD-*Parkin* group and GU-EOPD group.

	**PD-*Parkin n* = 24**	**GU-EOPD *n* = 24**	**P_**1**_**	**P_**2**_**
PDQ-39	22.48 ± 13.26	13.03 ± 9.76	0.007^**^	0.045^*^
**Domains**
Mobility	20.87 ± 14.95	9.27 ± 11.65	0.004^**^	0.025^*^
Activity of daily living	10.07 ± 10.63	14.93 ± 16.34	0.231	0.128
Emotional well-being	37.03 ± 22.74	17.54 ± 16.71	0.007^**^	0.015^*^
Stigma	34.90 ± 25.02	23.96 ± 22.01	0.115	0.187
Social support	16.84 ± 21.37	7.64 ± 14.52	0.089	0.277
Cognitions	19.27 ± 17.67	10.16 ± 10.87	0.038^*^	0.322
Communication	14.24 ± 17.97	9.72 ± 13.16	0.326	0.732
Bodily discomfort	27.78 ± 21.80	12.50 ± 11.53	0.005^**^	0.016^*^

*P_1_, comparison between PD-Parkin and GU-EOPD. P_2_, comparison between PD-Parkin and GU-EOPD after adjustment for age of onset and disease duration*.

* p < 0.05;

** *p < 0.01. GU-EOPD, Genetically undefined early-onset Parkinson's disease; PD, Parkinson's disease; PDQ-39, Parkinson's Disease Questionnaire-39*.

We then investigated the factors affecting the QoL in PD-*Parkin* patients and GU-EOPD patients respectively. The correlation analysis revealed that NMSQ (*p* = 0.001), BDI (*p* < 0.001), and ESS scores (*p* = 0.002) were positively correlated with PDQ-39 in PD-*Parkin* patients (Table 3). These significant relevant factors of PDQ-39 were then included in the stepwise multiple linear regression, and the result found that BDI scores (standardized β = 0.520, *p* = 0.005) were the strongest determinant of QoL in *Parkin*-related PD, followed by ESS scores (standardized β = 0.346, *p* = 0.047) (Table 4). In GU-EOPD, gender (*p* = 0.047), dystonia (*p* = 0.003), NMSQ (*p* = 0.001), and BDI scores (*p* < 0.001) were correlated with PDQ-39 scores (Table 3). The result of stepwise multiple linear regression found that BDI scores (standardized β = 0.860, *p* < 0.001) were the only determinant of QoL in GU-EOPD (Table 4).

**Table 3:** Correlated factors of QoL in PD-*Parkin* and GU-EOPD patients.

	**PD-*****Parkin***		**GU-EOPD**	
	***r***	***p***	***r***	***p***
Gender	0.141	0.510	0.410	0.047^*^
Age	−0.395	0.056	−0.218	0.305
Education	−0.113	0.609	−0.277	0.191
AOO	−0.221	0.299	−0.261	0.217
Disease duration	−0.044	0.839	0.133	0.537
LEDD	−0.255	0.230	−0.046	0.831
Dopaminergic agonist usage	0.014	0.949	0.269	0.204
Dystonia	0.229	0.282	0.580	0.003^**^
Motor fluctuations	0.182	0.396	0.233	0.273
Dyskinesias	0.193	0.366	NA	NA
UPDRS III	0.247	0.244	−0.015	0.944
H&Y	0.327	0.119	0.287	0.173
NMSQ	0.619	0.001^**^	0.743	<0.001^**^
BDI	0.682	<0.001^**^	0.860	<0.001^**^
EES	0.589	0.002^**^	0.042	0.848
RBDQ	0.376	0.070	−0.240	0.269
MMSE	−0.395	0.056	−0.403	0.057

* p < 0.05;

** *p < 0.01. AOO, Age of onset; BDI, Beck depression inventory; H&Y stage, Hoehn and Yahr stage; LEDD, Levodopa equivalent daily dosage; MMSE, Mini-mental state examination; NA, not applicable; NMSQ, Non-motor symptoms questionnaire; PD, Parkinson's disease. UPDRS III, Unified Parkinson's Disease Rating Scale part III*.

**Table 4:** Main determinants of QoL in PD-*Parkin* and GU-EOPD patients.

	**Standardized β**	***P*-value**
***Parkin*****-PD, adjusted** ***R***^**2**^ **=** **0.516**		
BDI	0.520	0.005^**^
ESS	0.346	0.047^*^
**GU-EOPD, adjusted** ***R***^**2**^ **=** **0.728**		
BDI	0.860	<0.001^**^

* p < 0.05;

** *p < 0.01. BDI, Beck depression inventory; ESS, Epworth sleepiness scale*.

## Discussion

We investigated the QoL and the influence factors of QoL in newly diagnosed PD patients with *Parkin* mutations by comparing them with GU-EOPD patients. We found PD-*Parkin* patients had younger AOO, longer disease duration, and tended to have a higher risk of dystonia. They also presented with more severe depression and EDS, along with worse scores in the NMSQ domains of depression/anxiety and sleep disorders. The QoL was much more reduced in PD-*Parkin*, with the subscales of mobility, emotional well-being, and bodily discomfort affected. BDI and ESS scores were found to be the significant determinants of QoL in PD-*Parkin* patients.

According to the literatures, few studies have focused on QoL in PD patients with *Parkin* mutations. Previous studies have compared the QoL between EOPD and late-onset PD (LOPD) patients and found the QoL of EOPD patients were worse than that of LOPD patients (9, 10). The AOO might have a great impact on the QoL since the younger patients tend to experience more frequent loss of employment, family life disruptions, depression and motor complications (18). Besides, some studies before investigated the QoL in different monogenic PDs as a group, comparing with idiopathic PD. However, the results were controversial (19, 20). Though the scales for QoL were different among those studies, the inconsistent results should be contributed to the genetic heterogeneity of the monogenic group. For the diversity of clinical characteristics in different genetic entities, it would be better to investigate QoL in patients with certain mutations. Therefore, we investigated life quality in the *Parkin*-related PD as it is one of the most common monogenic forms of PD (2). Since the prevalence of *Parkin*-related PD in patients with onset age after 40 years old was very low, so we chose the GU-EOPD patients with AOO <40 as the control, whose AOO might be similar to PD-*Parkin* group, to minimize the influence of age on the QoL. On the other hand, only patients with a diagnosis within 12 months were included to better interpret the effect of genotype on QoL without the interference of motor complications and other advanced comorbidities. Furthermore, one of the intended targets of this study was to offer evidence on the accurate management of QoL at the early stage of PD. Regrettably, the first-degree relatives of the patients as well as healthy subjects were not included in the study, which should be a goal in future studies.

Younger AOO, longer disease duration and common dystonia observed in our PD-*Parkin* patients were consistent with previous reports (3–5). Our data also revealed that late diagnosis was a prominent problem in PD-*Parkin*, with a mean disease duration of 6.75 years. The previous literature has drawn the same conclusion that significantly diagnostic delay was observed in *Parkin*-related PD, comparing with genetically undefined PD (21). Common diagnostic delay in *Parkin*-related PD was associated with the specific phenotype including the younger-onset age, lack of tremor, lower limb dystonia affecting gait, and slow progression (21, 22). Though the disease duration was statistically corrected in this study, the impact of long term untreated parkinsonian symptoms should still be considered as a contributor to poor life in PD-*Parkin*.

NMSs were less investigated in *Parkin*-related PD previously, and our result suggested that they suffered more NMSs burden. Though PD-*Parkin* showed the similar cognitive performance to GU-EOPD in objective assessments, they seemed to perform worse in the cognition domain of PDQ-39, which was a subjective assessment. Actually, the PDQ-39 cognitions scale was significantly associated with depression, instead of objective neuropsychological tests (23). And PD-*Parkin* did suffer more depression, as well as sleep disturbances, especially increased EDS. *Parkin* variants were considered as an important predictor of depressive symptoms (16, 24), and some PD-*Parkin* patients even self-reported depression 4–5 years exceeding motor onset, but none of them received the anti-depression treatment before. However, few studies focused on sleep problems of *Parkin*-related PD. One study stated that sleep measures including RBDQ and ESS were similar between the PD-*Parkin* and idiopathic PD (25). The result was somewhat contradictory to our findings. One possible explanation could be that the age of the idiopathic PD population was older than ours, ranging from 51 to 65 years. EDS was known as one of the most frequent sleep disorders in PD. Usually, male gender, older age, nighttime sleep problems, and dopaminergic agonist usage were related to increased risk of EDS in PD (26, 27). However, there were other studies indicating that apart from dopaminergic agonist, larger levodopa dosage was more significantly associated with EDS (26, 28, 29). Neither dopaminergic agonist nor levodopa usage differed between groups and both groups used a low dose of levodopa. Thus, we assumed that the higher ESS scores in PD-*Parkin* were more related to the specific genotype instead of medications.

We found worse QoL in PD-*Parkin* patients, especially in mobility, emotional well-being, and bodily discomfort. Worse mobility may be associated with motor symptoms and motor complications of PD-*Parkin*. Dystonia was only found as a significant relevant of QoL in GU-EOPD but not in PD-*Parkin*, which may resulted from the limited small sample size. However, since lower limb dystonia was so common in PD-*Parkin*, it still might be a contributor to the worse mobility in our PD-*Parkin* patients since it was recently reported to be associated with gait abnormalities (30), which were one of the most significant relevant factors to QoL in PD (31). As for the motor complications, motor fluctuations was significantly affected both PDQ-39 total scores and the dimension of mobility according to a large-scale cross-sectional study (32). In our study, a trend of higher prevalence of motor fluctuations was observed in PD-*Parkin* patients, which was likely to be another aspect accounting for the impaired mobility and QoL. Comparing with motor symptoms, NMSs played a more important role in reduced QoL (31, 33). A strong positive correlation was observed between the overall NMSs burden and PDQ-39 scores, especially the dimension of emotional well-being (11). Among the NMSs, depression and sleep disorders were correlated with worse QoL (11, 31, 33), and worse emotional well-being as well (33). Furthermore, it was reported that PD patients with EDS showed worse scores in all PDQ-39 subdomains (34). Notably, sleep disorders including EDS accompanied by depressive symptoms in many PD patients (35). Thus, EDS can work along with depression in worsening the QoL in PD-*Parkin*. Based on these results, the decreased QoL and its domain of emothinal well-being in PD-*Parkin* patients in the current study could be explained by the depression and sleep disorders. Besides, higher BDI scores were significantly associated with reduced threshold and tolerance of pain (36), which may result in higher scores in the domain of bodily discomfort.

We found depression was the determinant of QoL in both PD-*Parkin* and GU-EOPD patients while ESS was the other determinants of the QoL only in PD-*Parkin* patients. These findings underlined that attention should be paid to daytime sleepiness besides depression in patients with *Parkin* mutations, but these two symptoms were frequently underdiagnosed and undertreated by clinicians (37, 38). Careful history collection and routine screen for depression and EDS were recommended in *Parkin*-related PD. As for treatment, dopaminergic agonist pramipexole and Venlafaxin were recommended for depression treatment in PD (39), but clinicians should also be aware that the usage of dopaminergic agonist will probably increase the risk of EDS. Thus, whether to expand on depression treatment should be individualized, depending on which aspect was the dominant factor affecting the QoL. A balance should be achieved between depression treatment and excessive daytime sleepiness management. So Far, few pharmacological options were now available for EDS, and non-pharmacological therapies can be the main alternative in the management of EDS. Cognitive-behavioral therapy, light therapy, and repetitive transcranial magnetic stimulation can be optional approaches to manage EDS (40).

Several limitations of our study should be admitted. Firstly, this is a study of small sample size due to the relatively low prevalence of early-onset PD and *Parkin*-related PD. Large-scale and longitudinal studies are required to confirm our findings. Furthermore, though any pathogenic, likely pathogenic or risk variants in known PD-associated genes were ruled out in the GU-EOPD group, there is still potential heterogeneity in genetic background of the EOPD group. Additionally, the NMSs in our study were assessed by NMSQ, but not Non-Motor Symptoms Scales (NMSS). Therefore, the further detection of the effects of NMSs on QoL in *Parkin*-related PD was limited. Finally, though disease duration was statistically corrected, non-motor scores and QoL scores may not necessarily progress with disease duration in a linear way, which can result in erroneous conclusions.

## Conclusion

Worse QoL was observed in newly diagnosed PD-*Parkin* patients comparing with the GU-EOPD patients, with mobility, emotional well-being and bodily discomfort affected. Depression and EDS predicted worse QoL in *Parkin*-related PD. For clinicians, prompt recognition and accurate treatment of depression and EDS are important in the management of QoL in *Parkin*-related PD.

## Data Availability Statement

The original contributions generated for this study are available upon request to the corresponding author.

## Ethics Statement

The studies involving human participants were reviewed and approved by the Human Studies Institutional Review Board, Huashan Hospital, Fudan University. The patients/participants provided their written informed consent to participate in this study.

## Author Contributions

X-YZ and F-TL were involved in the drafting of manuscript and statistical analysis with design and execution. CC, S-SL, JZ, Y-LT, BS, and W-BY were involved in the data collection and review of the manuscript. C-TZ, J-JW, and Z-TD were involved in organization and execution of the project and revision of the manuscript. Y-MS and JW were involved in the conception, planning and supervising the execution of the research project, and critical revision final review of the manuscript. All authors contributed to the article and approved the submitted version.

## Conflict of Interest

The authors declare that the research was conducted in the absence of any commercial or financial relationships that could be construed as a potential conflict of interest.
