# Supplementary material for: Quality of Life in Newly Diagnosed Patients With Parkin-Related Parkinson's Disease
Source: Front Neurol. 2020 Dec 18;11:580910. doi: 10.3389/fneur.2020.580910 (PMC7775523; doi:10.3389/fneur.2020.580910)
Supplement: Supplementary file 1 [file Table_1.DOCX]

**Supplementary table 1** The genes tested in the patients.

| *ADCY5* | *ATXN3* | *GBA* | *MAPT* | *PDYN* | *SLC2A1* | *TGM6* |
| --- | --- | --- | --- | --- | --- | --- |
| *ADH1C* | *CIZ1* | *GCH1* | *MCCC1* | *PINK1* | *SLC6A3* | *TH* |
| *ANO3* | *DNAJC6* | *GIGYF2* | *MR1* | *PLA2G6* | *SNCA* | *THAP1* |
| *ATP13A2* | *DRD2* | *GNAL* | *PACRG* | *PRKRA* | *SPR* | *TOR1A* |
| *ATP1A3* | *EIF4G1* | *HTRA2* | *PANK2* | *PRRT2* | *SYNJ1* | *TUBB4A* |
| *ATP6AP2* | *FA2H* | *IL1B* | *PARK2* | *RAB39B* | *TAF1* | *UCHL1* |
| *ATXN2* | *FBXO7* | *LRRK2* | *PARK7* | *SGCE* | *TBP* | *VPS35* |
