# Supplementary material for: Quality of Life in Newly Diagnosed Patients With Parkin-Related Parkinson's Disease
Source: Front Neurol. 2020 Dec 18;11:580910. doi: 10.3389/fneur.2020.580910 (PMC7775523; doi:10.3389/fneur.2020.580910)
Supplement: Supplementary file 2 [file Table_2.DOCX]

**Supplementary Table 2** Genetic variants detected in the PD patients with *Parkin* mutations

| Patient No. | Allele 1 | ACMG evaluation | Allele 2 | ACMG evaluation |
| --- | --- | --- | --- | --- |
| 1 | exon 3-5 deletion | - | c.850G>C | - |
| 2 | exon 2 deletion | - | exon 3 deletion | - |
| 3 | exon 3 deletion | - | exon 3 deletion | - |
| 4 | exon 4 deletion | - | exon 4 deletion | - |
| 5,6 | exon 7 deletion | - | exon 7 deletion | - |
| 7 | exon 7 deletion | - | c.850G>C | - |
| 8 | exon 3 deletion | - | c.98G>A & exon 7 deletion | - |
| 9 | c.79_81del* | PM1; PM3; PM4; PP4 | c.79_81del* | PM1; PM3; PM4; PP4 |
| 10 | c.823C>T | - | c.823C>T | - |
| 11 | exon 3-4 deletion | - | c.1321T>C* | PM1; PM2; PM3; PP3; PP4 |
| 12 | exon 2-4 deletion | - | exon 6 deletion | - |
| 13,14 | exon 3 deletion | - | c.1321T>C* | PM1; PM2; PM3; PP3; PP4 |
| 15 | exon 4 deletion | - | exon 6 deletion | - |
| 16 | exon 2-4 deletion | - | exon 5-6 duplication | - |
| 17, 18 | exon2-3 deletion | - | exon2-3 deletion | - |
| 19 | exon 2 deletion | - | exon3-4 deletion | - |
| 20 | c.850G>C | - | c.1385G>A | - |
| 21 | exon 7 deletion | - | c.1010G>A* | PM1; PM2; PM3; PP3; PP4 |
| 22 | exon 3 deletion | - | exon 4 deletion | - |
| 23 | exon 6-7 duplication | - | exon 6-7 duplication | - |
| 24 | exon 6-7 deletion | - | exon 6-7 deletion | - |

Transcripts: ***Parkin*:** NM_004562.

**^*^**, rated as likely pathogenic according to the ACMG guideline.

“-“ in the ACMG column means the variant is confirmed a mutation according to the literatures or ClinVar (https://www.ncbi.nlm.nih.gov/clinvar/)
