# Supplementary figures and images for: Quality of Life in Newly Diagnosed Patients With Parkin-Related Parkinson's Disease
Source: Front Neurol. 2020 Dec 18;11:580910. doi: 10.3389/fneur.2020.580910 (PMC7775523; doi:10.3389/fneur.2020.580910)

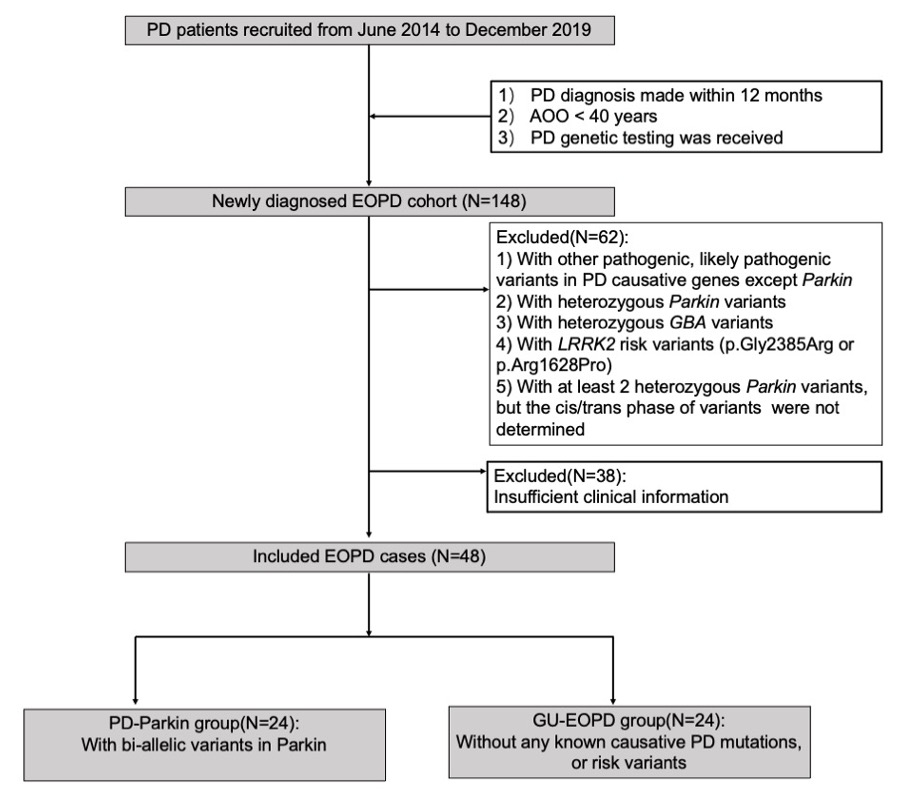

Supplement: Supplementary file 3 [file Image_1.jpeg]
